# Inequality in geographical distribution of hospitals and hospital beds in densely populated metropolitan cities of Iran

**DOI:** 10.1186/s12913-019-4443-0

**Published:** 2019-08-30

**Authors:** Yousef Chavehpour, Arash Rashidian, Abraha Woldemichael, Amirhossein Takian

**Affiliations:** 1 Department of Health Management and Economics, School of Public Health, Tehran University of Medical Sciences, Tehran, Iran; 2 School of Public Health, College of Health Sciences, Mekelle University, Mekelle, Ethiopia; 3 Department of Global Health and Public Policy, School of Public Health, Tehran University of Medical Sciences, Tehran, Iran; 4 Health Equity Research Centre (HERC), Tehran University of Medical Sciences, Tehran, Iran

**Keywords:** Geographical distribution, Inequality, Hospitals, Hospital beds, Main cities, Regionalization

## Abstract

**Background:**

This study aims to assess geographical distribution of hospitals and extent of inequalities in hospital beds against socioeconomic status (SES) of residents of five metropolitan cities in Iran.

**Methods:**

A cross-sectional analysis was conducted to measure geographical inequality in hospital and hospital bed distributions of 68 districts in five metropolitan cities during 2016 using geographic information system (GIS), and Gini and Concentration indices. Correlation analysis was performed to show the relationship between the SES and inequality in hospital beds densities.

**Results:**

The study uncovered marked inequalities in hospitals and hospital beds distributions. The Gini indices for hospital beds were greater than 0.55. The aggregated concentration indices for public and private hospital beds were 0.33 and 0.49, respectively. The GIS revealed that 216 (70.6%) hospitals were located in two highest socioeconomic status classes in the cities. Only 29 (9.5%) hospitals were located in the lowest class. The public, private, and the cumulative hospitals beds distributions in Tehran and Esfahan showed significant (*p* < 0.05) positive correlation with SES of the residents.

**Conclusions:**

The high inequalities in hospital and hospital beds distributions in our study imply an overlooked but growing concern for geographical access to healthcare in rapidly urbanizing metropolitan cities in Iran. Thus, regardless of ownership, decision-makers should emphasize the disadvantaged areas in metropolitan cities when need arises for the establishment of new healthcare facilities in order to ensure fairness in healthcare. The metropolitan cities and rapid urbanization settings in other countries could learn lessons to reduce or prevent similar issues which might have hampered access to healthcare.

## Background

The concern for equitable healthcare access to people is a fundamental element of many health policies. Governments have been committed to ensuring access to healthcare, and success is highly dependent on the nature of existing inequality and in the healthcare needs of people [1]. Thus, access to healthcare is a multidimensional concept that is subject to the influences of both the geographical, socio-demographic [2], information, and economic factors. The service availability is another influencing factor for the geographical accessibility of healthcare [3].

Studies reported differences in the geographical distribution of healthcare facilities based on the socioeconomic status [4, 5] and location of residential areas in a city where suburban residents had lower accessibility to healthcare facilities than residents in the central city [6]. Evidence from a recent study in China indicates extremely high inequalities in the geographical distribution of health facilities, health workforce, and hospital beds [7]. Regardless of the socioeconomic status (SES) and other distinguishing parameters of people, providing an opportunity of equal access to basic healthcare services is essential for ensuring justice in healthcare [8, 9]. However, this is not an easy task, especially in the under-resourced countries where the inequalities in the distribution of healthcare infrastructure and health workforce are common challenges [10].

The provision of healthcare often follows an inverse care law. This law completely operates where medical care is exposed to market forces. In areas with fewer healthcare facilities, more diseases and deaths are likely to happen. The phenomena are more pertinent among the low SES communities [11]. Geographical access to healthcare services contribute to the minimization of the health inequalities [12], and there existed an inverse relationship between geographical access to a healthcare facility and healthcare utilization [13, 14]. Therefore, measuring the locations of hospitals relative to the residents in different SES can provide a clear picture of the geographical inequalities in hospital distributions [15–17].

Iran’s healthcare system is organized into national, provincial, and city levels [18], and is primarily an insurance-based [19]. The focus of the healthcare system has been on primary care with a clearly defined vision and strong government commitment that demonstrated success in realizing the universal access to health care [20]. However, the issue of marginalization and increasing slum areas due to urbanization and high rural-urban migration challenged the equitable access to healthcare [21]. Despite the reports of few studies on healthcare and distributions of health facilities in the urban and rural settings in Iran [22, 23], the issues of inequalities in hospitals and hospital bed distributions in the highly populated cities received less attention.

A recent study analyzed inequality in the distribution of hospitals and assessed the inverse care law hypothesis. The findings revealed high inequalities in the geographical distributions of hospitals and hospital beds in favor of the relatively affluent areas [24]. The study provided a new way of viewing and measuring inequalities in metropolitan cities. The current study is a continuation of the previous one on a wider scale to determine the extent of the inequalities in the hospitals and hospital beds with respect to the SES of residents in five metropolitan cities in Iran. Measuring socio-economic inequalities in hospital and hospital beds help informed policy decisions to ensure equity in healthcare in the urban settings of Iran and perhaps other similar contexts.

## Methods

### Setting

Iran is an upper-middle-income country having a total population of about 80 million people inhabiting in the country’s 31 provinces, out of which 74% resides in the urban settings. The total number of all type services providing hospitals during 2016 in Iran was 921. Both teaching and non-teaching governmental (public), private, social security organization (SSO), military, and charity hospitals accounted for 568, 161, 74, 52, and 30, respectively. The remaining 36 were affiliated with other non-public organizations. A quarter (25%) of the total population lives in eight top largest cities namely Tehran, Mashhad, Esfahan, Karaj, Shiraz, Tabriz, Qom, and Ahvaz [25]. This study purposefully included Tehran, Mashhad, Esfahan, Shiraz and Tabriz metropolitan cities, and are located in the northern, eastern, central, southern, and western parts of Iran. Their geographical distributions fairly represent the metropolitan cities in Iran. A summary of the total number of districts, populations, and total hospitals beds within each city is presented in Table 1.

**Table 1:** Summary of districts, populations, hospitals and hospital beds distributions by metropolitan city in Iran

Item	Mashhad	Tabriz	Shiraz	Esfahan	Tehran
Districts	13	10	9	14	22
Total population (in number)	3,001,184	1,558,693	1,565,572	1,961,260	8,693,706
Total hospitals	39	29	40	36	162
Hospital beds					
Private	1099	803	1026	689	7488
Public	6769	4995	5488	5107	22,719
Total	7868	5798	6544	5796	30,207

### Study design and data source

We measured the inequality in the distributions of hospitals and hospital beds using different techniques in five metropolitan cities during 2016 in Iran. The dataset consisted of hospitals, hospital beds, populations, and residential floor area per capita for a total of 68 districts of the cities. We retrieved the data on total population and residential floor area per capita of the districts’ residents from the Statistics Center of Iran (SCI). The data on the number and geographical locations of the functioning hospitals in the districts and hospital beds for the year 2016 were obtained from the municipalities of the cities, the hospitals’ database, the Ministry of Health and Medical Education (MOHME) of Iran, and the National Cartographic Centre of Iran.

### Variables

The total hospitals (both governmental, private, and other public organization affiliated hospitals) providing services in each district of the cities were included in the study. The number of hospital beds per 10,000 population [26, 27] was the variable (indicator) used to measure the inequality of access to hospitals. Nevertheless, the residents are more likely to form and maintain a system of social stratification along multiple dimensions including socio-economic status [28]. The most commonly used socio-economic status (SES) indicators in health care research are educational status, income, wealth, housing condition with its location, overcrowding (residential density), etc. [28–33].

The area level SES indicators are useful not only to characterize the extent of inequality in resources distributions but also as a measure of the SES of individuals. For example, overcrowding (> 1 person in a room) often implies few economic resources of the household [29, 34]. Therefore, our analysis applied residential floor area per capita (m^2^/person) to measure the socio-economic inequality in hospitals and hospital beds because there was no data on income per capita of the people. Despite income per capita is a direct measure of SES [28], the residential floor area per capita is a comprehensive indicator of the economic, social, cultural, and environmental dimensions of people living in a given urban area [35, 36].

### Analysis and interpretation of inequality

In this analysis we categorized the hospitals into public including the SSO hospitals, private, and other ownership hospitals such as military, charity, etc. The descriptive analysis and the mapping of the geographical locations of hospitals using the Geographic Information System (GIS) were based on all 306 hospitals. The inequality measures were based on the public and private hospitals, and the unit of the analysis was district. We applied different methods of analysis and characterized the distributions using the district level data of five metropolitan cities in Iran. First, we used the administrative boundaries of the districts as reference points for identifying the locations of the hospitals and residential areas. Then, the hospitals were mapped against the floor area per capita of the residents using the Geographic Information System (GIS) in QGIS 3.8 software to have an insight about the geographical locations of the hospitals with respect to the SES of the residents [37, 38]. The QGIS is an open source software for mapping spatial data. The natural break [39] was used to categorize and display the categories of the residents’ access to hospitals against their SES, and the output maps have been illustrated using the QGIS.

Second, we used the Gini and concentration indices to measure the inequalities in the distribution of the hospital beds. The Gini index (GI) is a standard measure of distributional inequalities in healthcare with respect to population size and has a direct relationship with the Lorenz curve [26]. It is an important measure when the fairly aggregated distributions involve relatively few and large geographic units and is mathematically described as [40]:

$$ GI=1-{\sum}_{i=1}^{k-1}\left({Y}_{i+1}+{Y}_i\left)\right({X}_{i+1}-{X}_i\right) $$

Where **Y**, is the cumulative proportion of hospital beds per 10,000 people over **k** districts, and **X** is the cumulative proportion of the population ranked by floor area per capita.

The Gini values range from 0 (perfect equality) to 1 (perfect inequality). Despite the degree of the Gini inequality is context specific and may be interpreted in different ways, our analysis interpreted the extent of the inequality based on the five scale values categorized as absolute equality (GI < 0.2), high equality (GI = 0.2–0.3), inequality (GI = 0.3–0.4), high inequality (GI = 0.4–0.6], and absolute inequality (GI > 0.6) [26, 41].

We further applied the concentration index (CI) to measure the socioeconomic inequality of hospital beds distributions [7]. The CI can be defined as twice the area between the concentration curve and the 45-degree line of equality, and highlights the extent of unfair inequality which is amenable to policy or action [42, 43]. The CI can be calculated the same way as the GI but the value always lies in the range [− 1, + 1]. The values of CI < 0, CI = 0, and CI > 0 indicate the disproportionate concentration of the distribution in favor of the poor, proportionate distribution, and disproportionate concentration of the distribution in favor of the rich, respectively. The more the value deviates away from 0, the higher is the inequality [43, 44]. The Gini and concentration indices were calculated using the latest official Stata command “*conindex*”. This command provides the inequality measured value with its standard error and *p*-value, and is an appropriate estimate of the rank-dependent indices of univariate inequality [43].

Finally, correlation analysis was performed to show the relationship between public, private and total hospital beds distributions and residential floor area per capita for each metropolitan city. The analyses were done using the statistical software package STATA 13 and considered significant at *p*-value less than 0.05.

## Results

This study analyzed the relationship between residential floor area per capita and locations of hospitals using QGIS, the extent of Gini inequality in hospital beds distributions, and the extent of socio-economic inequality in hospital beds distribution in five metropolitan cities in Iran. Out of the total 306 hospitals included in the analysis, the public, private and other hospitals accounted for 128 (41.8%), 101 (33.0%), and 77 (25.2%) hospitals, respectively. Sixty (46.9%) of the public hospitals, 62(61.4%) of the private hospitals, and 40 (51.9%) other ownership hospitals found in Tehran. The rest hospitals were in the remaining four cities. The aggregated hospital bed to population ratio of the five cities was 1:32 (that is, 32 beds per 10,000 population). The average hospital beds to population ratios were 1:35 in Tehran, 1:42 in Shiraz, 1:37 in Tabriz, 1:30 in Esfahan, and 1:26 in Mashhad, respectively (Table 1).

**Fig. 1:**
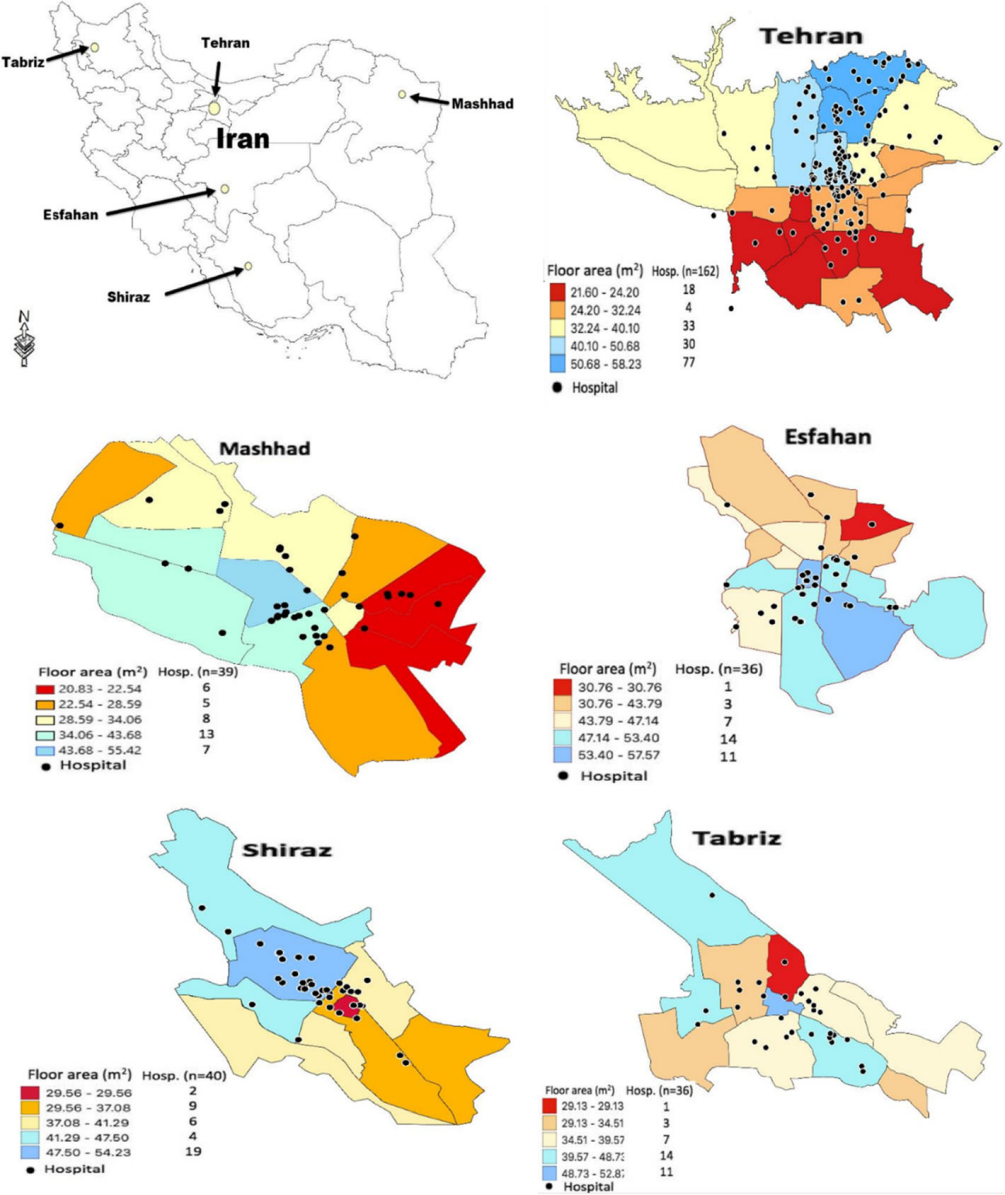
Geographical location of hospitals against residential floor area per capita in districts of five metropolitan cites in Iran (sources: authors work)

The hospitals distributions are in favor of districts with larger residential floor area per capita (Fig. 1). For example, 66% (107/162) of the hospitals in Tehran and 51.3% (20/39) in Mashhad are located in the districts ranked in two highest socioeconomic classes. Similarly, the rates for Tabriz, Shiraz and Esfahan were 37.9% (11/29), 57.5% (23/40) and 69.4% (25/36), respectively. The GIS revealed that 216 (70.6%) hospitals are located in the two highest socioeconomic classes in the cities. Only 29 (9.5%) hospitals are located in the lowest class. The SES of the residents is presented in categories ranging from poor (deep red color) to rich (light-blue color) as shown in Fig. 1.

Regardless of ownership, the Gini indices (GIs) for the hospital beds in all the cities were above 0.55 (Table 2). The concentration indices for the private hospital beds in Mashhad and Tehran, and for the public hospital beds in Esfahan and Tehran were significantly high (*p* < 0.05). The aggregated CI value for the private hospital beds per capita (CI = 0.49) was insignificant (*p* = 0.059), while for the public hospitals beds (CI = 0.33) and the total hospital beds (CI = 0.29) per capita were significant (*p* < 0.05) (Table 3).

**Table 2:** Gini and concentration indices for hospital beds distributions in five metropolitan cities in Iran (* *p* value < 0.05)

Hospital beds	Indices	Main Cities				
		Mashhad	Tabriz	Shiraz	Esfahan	Tehran
Private hospital beds	GI (S.E)	0.79 (0.10)*	0.76 (0.17)*	0.80 (0.30)*	0.68 (0.15)*	0.80 (0.19)*
	CI (S.E)	0.60 (0.26)*	0.28 (0.35)	0.73 (0.37)	0.34 (0.20)	0.61 (0.24)*
Public hospital beds	GI (S.E)	0.66 (0.11)*	0.74 (0.21)*	0.72 (0.23)*	0.63 (0.10)*	0.67 (0.12)*
	CI (S.E)	0.32 (0.21)	0.51 (0.31)	0.35 (0.34)	0.52 (0.15)*	0.37 (0.17)*
Total hospital beds	GI (S.E)	0.57 (0.15)*	0.63 (0.15)*	0.66 (0.17)*	0.56 (0.07)*	0.64 (0.12)*
	CI (S.E)	0.34 (0.21)	0.34 (0.25)	0.25 (0.29)	0.45 (0.12)*	0.39 (0.17)*

**Table 3:** Aggregated concentration indices for public, private and total hospitals beds of five metropolitan cities in Iran

Variable	C.I (S.E)	*p*- value
Private hospital beds	0.49 (0.19)	0.059
Public hospital beds	0.33 (0.04)	0.001
Total hospital bed	0.29 (0.06)	0.009

**Fig. 2:**
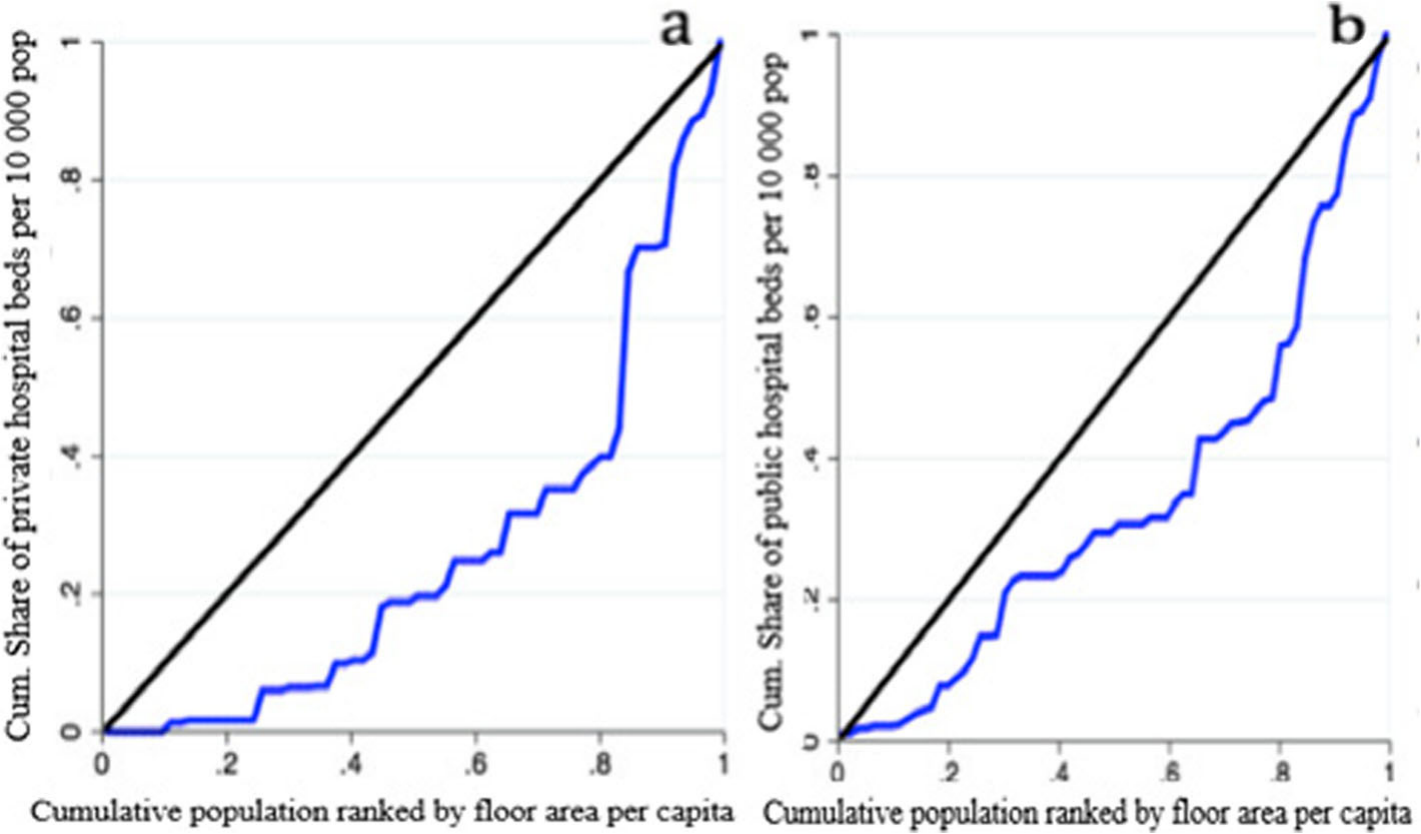
Concentration curves for (**a**) private, and (**b**) public hospital beds in five metropolitan cities in Iran

About 25% of the residents in the lowest rank of the residential floor area per capita have almost no access to the private hospital beds, while 20% of people in the highest rank of the residential floor area per capita have access to 60% of the private hospital beds (Fig. 2a). Furthermore, around 20% of people in the lowest rank of the residential floor area per capita have access to about 5% of the public hospital beds, whereas 20% of residents in the highest rank of the floor area per capita have access to about 50% of the hospital beds (Fig. 2b).

The geographical distribution of the public, private and total hospitals beds in Tehran revealed moderately strong (*p* < 0.05) positive correlation with the SES of the residents. In Esfahan, the public hospital beds and the overall hospitals beds distributions had strong positive correlation (p < 0.05), while the private hospitals beds distributions in Mashhad and Shiraz were strongly (*p* < 0.05) positively correlated (Table 4).

**Table 4:** Correlation between public, private, and all hospitals beds distributions with socioeconomic status of residents of districts in five metropolitan cities in Iran

City	Hospital beds	Correlation	*P*-value
Tehran	Public	0.48	0.022
	Private	0.54	0.009
	Total	0.52	0.013
Mashhad	Public	0.49	0.086
	Private	0.61	0.026
	Total	0.44	0.134
Esfahan	Public	0.61	0.021
	Private	0.45	0.017
	Total	0.67	0.008
Shiraz	Public	0.55	0.122
	Private	0.74	0.022
	Total	0.46	0.207
Tabriz	Public	0.43	0.209
	Private	0.15	0.689
	Total	0.41	0.237

## Discussion

This study analyzed the geographical distribution of hospitals and hospital beds in relation to socioeconomic status of districts in five metropolitan cities in Iran. Different approaches were used to measure and characterize the inequalities in hospital beds distributions. The GIS indicated that the majority of the hospitals are located in districts with relatively higher socioeconomic status. Other studies also reported inequitable distribution of hospitals in urban settings [2, 38, 45] and a significant difference between public and private health facilities distributions [46]. This might imply an overlooked issue of inequality in healthcare and the financial and non-financial consequences it might have especially in highly populated cities [10, 47].

A study found inequalities in the distribution of health facilities based on socioeconomic status of people [45]. The establishment of hospitals in areas where the socioeconomically privileged people live like found in our study could have contributed to a negative influence in healthcare utilization and outcomes. This is because not only the geographical distance of the location of the hospitals but also in the highly populace cities that the travel-time to seek healthcare is influenced by high traffic [48]. This situation can lead to delay in medical diagnosis, especially among the socioeconomically disadvantaged population groups [49] and to a high cost of travel to seek healthcare among the service users.

The Gini and concentration indices also revealed high inequalities in the distribution of hospital beds in the cities. The overall inequalities for hospital beds in our study are higher than those reported in another study [50]. However, the Gini values alone are not sufficient to inform policy about the actual inequality in healthcare [51]. Thus, the inequality in private hospital beds with respect to the floor area per capita of residents was significantly in favor of the rich in two (Tehran, and Mashhad) of the five main cities. This might imply that the private hospitals are being established in the districts with the richer people as they are for profit or the owners might have preferred areas that will increase their utility. Such differences might contribute to the worsening of the maldistribution of medical resources [11]. The inequalities in the public hospital beds were significantly in favor of the rich in only two (Tehran and Esfahan) of the five cities analyzed. The concentration curves of the hospital beds also revealed the tendencies to be in favor of the rich. Previous studies in Tehran and elsewhere also found hospital beds distributions and hospital visit per capita in favor of the rich [24, 52, 53].

Our study indicated a disproportionately higher disparity in the distribution of hospitals and hospital beds in the districts of the cities in favor of the rich. A study in Japan identified scarcity of healthcare resources in low socioeconomic population groups, an imbalance in healthcare services provision, and a positive correlation between hospital bed density and inpatient flow ratio [54]. An association between highway and major arterial roads, number of subway entrances, and row house areas with hospital distributions in a metropolitan city was also reported [55]. The different analytical approaches applied in our study clearly demonstrated the inequality in hospital and hospital beds concentrations. The inequalities in geographical distribution of the healthcare infrastructure, particularly the hospitals and hospital beds in metropolitan cities are usually mediated by a disproportionately operating multiple factors including the social, economic, infrastructure and other dimensions. Our study uncovered the extent of existing disparities in the distributions of hospitals and hospital beds in the metropolitan cities in Iran. The findings highlight not only the policy and level of effort required to address the identified gap in access to healthcare in the metropolitan cities but also inform the strategies to be followed to ensure fair access of hospital services and prevent the occurrence of similar issue in the growing urban settings in Iran.

The study has some important limitations. The use of residential floor area per capita is a valid and relevant measure of SES, however, it is not a direct indicator of SES of the residents. Identifying other determinants of access to healthcare such as road traffic, travel time, cost, etc. in the metropolitan cities could better inform policy decisions. This study was limited to few number of metropolitan cities in Iran, and the analysis did not consider the populations living proximal to the study cities, while it could have some effect to the measured inequalities.

## Conclusion

This study uncovered the presence of clear inequalities among the main cities in the distribution of hospitals and hospital beds in favor of people in the higher socioeconomic status. The inequalities were particularly high for the distributions of the private hospitals and hospital beds, and  indicates an overlooked but growing concern of access to healthcare in rapidly urbanizing countries like Iran. This implies when need arises for the establishment or opening of new healthcare facilities, regardless of ownership, decision-makers should emphasize to the disadvantaged urban districts in order to ensure fairness in healthcare. Our findings indicated the allocation of hospitals towards more affluent areas in the geographically representative metropolitan cities at national level. As such it is important that hospital establishment plans should not only consider the distributions among different cities, but also within geographical localities of major urban settings. This recommendation should be qualified with the expected limitations related to economies of scale and scope in hospital planning to ensure access to health care and efficiency considerations.

## Data Availability

The data that support the findings of this study are available from the Ministry of Health and Medical Education of Iran, Statistics Center of Iran (SCI), individual hospitals’ databases, and National Cartographic Centre of Iran. Restrictions apply to the availability of the complete data, which were used under agreement for the current study, and so are not all publicly available. Data are however available from the authors upon reasonable request and with permission of the mentioned organizations.

## Abbreviations

CI: Concentration Index
GI: Gini index
GIS: Geographic Information System
MOHME: Ministry of Health and Medical Education of Iran
SCI: Statistics Center of Iran
